# Development of a Novel Matrix Reference Material for Four Bisphenols in Fish Muscle Powder

**DOI:** 10.3390/foods15172986

**Published:** 2026-08-25

**Authors:** Yunjia Yang, Xiao Xu, Qian Jiang, Jing Zhang, Jie Yin

**Affiliations:** 1 Beijing Center for Disease Prevention and Control, Beijing 100013, China; 2 School of Public Health, Capital Medical University, Beijing 100069, China

**Keywords:** matrix reference material, bisphenol analogues, fish muscle powder, quality control

## Abstract

A novel matrix reference material (RM) for accurate determination of bisphenol A (BPA), bisphenol F (BPF), bisphenol S (BPS) and bisphenol AF (BPAF) in fish muscle was first developed. The RM candidate was prepared by intraperitoneal administration of target compounds to tilapia, followed by muscle mincing, homogenization, freeze-drying, milling and subpacking. Homogeneity, stability and characterization studies of the RM were carried out using an optimized isotope dilution-liquid chromatography tandem mass spectrometry (ID-LC-MS/MS) method. Sufficient homogeneity of the RM is suggested by ANOVA analysis. Short-term and long-term stability assessments, based on linear regression analysis, indicated no significant time-dependent drift for the RM stored at 60 °C for 10 days or at −20 °C for 12 months. From an interlaboratory study involving eight laboratories, the assigned indicative values of the RM were 8.71 μg/kg for BPA, 7.36 μg/kg for BPF, 10.10 μg/kg for BPS, and 5.43 μg/kg for BPAF, with corresponding expanded uncertainties (*k* = 2) of 0.71, 0.56, 0.80 and 0.42 μg/kg, respectively.

## 1. Introduction

Bisphenol A (BPA) is an important organic intermediate material for the industrial synthesis of polycarbonate and epoxy resins [1]. It is widely used in the production of chemical products such as plastics, coatings, and adhesives, as well as daily necessities such as food packaging materials, food can liners and paper products [2]. The extensive use of BPA has resulted in widespread human exposure to this compound through different routes, including oral intake, inhalation and dermal contact. For the general population, dietary intake has been considered as the major source of BPA exposure [1,2,3]. Previous experimental and epidemiological studies have linked low-level exposure of BPA to a variety of adverse effects on human health, such as cancer, infertility, diabetes and obesity [4]. Considering its potential health risks to humans, BPA usage has been restricted in many countries and districts, including the United States [5], Canada [6], the European Union [7], Switzerland [8] and China [9]. The European Food Safety Authority [4] has carried out extensive risk assessments on BPA since 2006, and established a tolerable daily intake (TDI) value of 0.2 ng/kg bw/day for BPA in 2023, which is twenty thousand times lower than its former TDI set in 2015.

With respect to these limitations, the industrial applications of BPA are gradually being replaced by its structural analogues, such as bisphenol F (BPF), bisphenol S (BPS) and bisphenol AF (BPAF) [2]. Unfortunately, these analogues do not seem to be safer than BPA. In vitro and in vivo studies have demonstrated that BPF, BPS and BPAF can exert endocrine disruption effects, cytotoxicity, neurotoxicity and reproductive toxicity similar to those of BPA [10,11]. Epidemiological studies revealed that low-level exposure to these BPA analogues may cause fetal growth restrictions, neurological dysfunctions and metabolic disorders [12]. On the other hand, these analogues can also be widely detected in a variety of foodstuffs, with relatively higher concentrations in aquatic foods [2,13]. Hence, as previously mentioned for BPA, dietary ingestion is also considered to be the primary pathway of human exposure to these analogues. However, compared with the extensive research on BPA, studies on the occurrence and toxicity of BPF, BPS and BPAF remain limited, and their potential health risks have not been fully identified.

Accurate and reliable analytical methods serve as the foundation for concentration monitoring, risk assessment and even the development of control measures for food contaminants [14,15]. To determine the levels of bisphenol A and its analogues in food matrices, different types of detection technologies have been used in previous studies, e.g., high-performance liquid chromatography (HPLC) with a fluorescence detector [16], liquid chromatography tandem mass spectrometry (LC-MS/MS) [17,18,19,20], gas chromatography with mass spectrometry (GC-MS) [21], surface-enhanced Raman scattering [22], electrochemical sensors [23] and biosensors [24]. Various sample pretreatment procedures, such as ultrasonic-assisted extraction [17,19,20,23], dispersive liquid–liquid microextraction [21], mixed matrix membrane-based extraction [16], QuEChERS [18,21], solid phase extraction (SPE) [17,20] and on-line SPE [19], have been established to enrich bisphenol analogues in foodstuffs and remove interference in complex matrices, which have important influences on the accuracy, precision and sensitivity of the entire analytical methods. However, it is difficult to ensure the reliability and comparability of measurement results generated by these diverse methods across different laboratories, because only fortified samples, rather than matrix reference materials (RMs), were applied for method validation and quality control. These in-house fortified samples are mainly tailored for matrix effect correction or routine batch recovery monitoring, without systematic homogeneity and stability assessment or formal value assignment. By contrast, matrix RMs are higher-order metrological materials with good homogeneity and stability. They possess matrix compositions identical to those of real samples, and have accurate and reliable characteristic values with uncertainty and traceability [25]. Such materials are indispensable measuring tools in laboratory method validation, proficiency tests and mutual recognition, and have been widely applied in food analysis to improve the accuracy of measurement results [14,26]. However, although the widespread contamination of bisphenol analogues is of great concern, matrix RMs for the determination of these compounds in food are still very limited.

The aim of our present study was to develop a matrix RM for BPA, BPF, BPS and BPAF in fish muscle, as aquatic foods have been found to be a potentially important contributor to human bisphenol exposure [13]. In this paper, we report on the process of obtaining large quantities of fish muscle samples containing bisphenols at low μg/kg levels by intraperitoneal injection of fish with target analytes. The processing of raw fish muscle to yield candidate powder materials is then described together with the homogeneity, stability and value assignment studies. Finally, we analyze and evaluate the uncertainties induced in the development of the matrix RM. To the best of our knowledge, this is the first report on a matrix RM for bisphenols in fish muscle.

## 2. Materials and Methods

### 2.1. Chemicals and Reagents

Certified stock solutions of BPA (100 μg/mL ± 1.5%, product no. GBW08754), BPF (100 μg/mL ± 2.0%, product no. GBW08755), BPS (100 μg/mL ± 1.5%, product no. GBW08756), and BPAF (100 μg/mL ± 1.9%, product no. GBW08757) in methanol were obtained from the National Institute of Metrology of China (Beijing, China) and used for LC-MS/MS analysis. The standards of the four analytes with purities greater than 98% were purchased from Tokyo Chemical Industry Co., Ltd. (Tokyo, Japan), and used for fish administration. The isotope internal standards of ^13^C_12_-BPA (product no. CLM-4325-1.2), ^13^C_12_-BPF (product no. CLM-9866-1.2), ^13^C_12_-BPS (product no. CLM-9319-1.2) and ^13^C_12_-BPAF (product no. CLM-9776-1.2) with concentrations of 100 μg/mL in acetonitrile or methanol were obtained from Cambridge Isotope Laboratories, Inc. (Tewksbury, MA, USA).

The methanol and acetonitrile used for sample pretreatment were of HPLC grade and were purchased from Dikma Technologies Inc. (Lake Forest, CA, USA). Ultrapure water for sample pretreatment was obtained from a Milli-Q ultrapure water system (Millipore Corporation, Billerica, MA, USA). The Methanol and water used for instrumental analysis were of LC-MS grade and were purchased from Honeywell (Morristown, NJ, USA). Formic acid with purity of 99% was supplied by Acros Organics (Waltham, MA, USA).

### 2.2. Preparation of the Candidate Reference Material

10 mg of each target bisphenol was weighed and dissolved in dimethyl sulfoxide (DMSO) to prepare stock solutions with concentrations of 10 mg/mL. The mixed standard solutions of four bisphenols with concentrations of 100 μg/mL BPA, 1500 μg/mL BPF, 100 μg/mL BPS and 25 μg/mL BPAF were prepared by mixing appropriate volumes of each stock solution and diluting with water. These mixed standard solutions were subsequently applied in fish administration to create incurred raw materials. All solutions were freshly prepared prior to fish administration, and good solubility and stability of the target bisphenols were confirmed after aqueous dilution of DMSO stock solutions.

Commercial edible tilapia (*Oreochromis niloticus*) weighing approximately 1 kg were purchased from a fixed local supermarket, and acclimated in water tanks at room temperature for 12 h without feeding. Next, each tilapia was intraperitoneally injected with the mixed standard solution of four bisphenols at doses of 100 μg/kg BPA, 1500 μg/kg BPF, 100 μg/kg BPS and 25 μg/kg BPAF based on its own weight, resulting in slight variations in practical injection volumes ranging from 0.9 to 1.1 mL per fish. After injection, the tilapia was continuously reared for 12 h, and then humanely euthanized through a 5 min ice-water bath followed by a blunt force trauma to the head. This procedure was fully compliant with EU Directive 2010/63 on the ethical standards for scientific animal use [27]. Positive muscle samples were dissected from both epaxial and hypaxial trunk musculature at multiple body segments, carefully separated from overlying skin, subcutaneous fat and the underlying vertebral column and intermuscular bones. The excised muscle tissues were then rinsed with ultrapure water to remove surface blood clots and adherent debris. In addition, blank muscle samples were collected from tilapia without administration. Details on tilapia husbandry and exposure experiments are provided in Supplementary Materials Text S1.

To prepare raw materials with expected concentrations, positive and blank muscle samples were separately cut into pieces of approximately 1 cm^3^ and homogenized into a fine paste at high speed. The resulting positive and blank homogenates were then mixed at a mass ratio of 1:9 and further homogenized thoroughly. After being prefrozen at −40 °C in a refrigerator, the fish muscle paste was loaded into a GZLY-5 freeze-dryer (Songyuan Huaxing Technology Develop Co., Ltd., Beijing, China). Lyophilization was performed with the shelf temperature programmed from −20 °C to 25 °C, a chamber pressure of 30 Pa, and a condenser temperature of −50 °C. The freeze-drying process was terminated when the temperature difference between the monitored sample and the shelf remained within 1 °C for 2 consecutive hours. The freeze-dried materials were then crushed and milled for 1 h at 25000 rpm using a YS-10 mill (Yanshan Zhengde Equipment Co., Ltd., Beijing, China). Thereafter, the materials were manually sieved through a 40-mesh stainless steel sieve (nominal aperture 425 μm) which was rinsed sequentially with methanol and ultrapure water before use, and then blended for 5 h in an HD50 three-dimensional mixer (Liming Machinery Company, Taizhou, China). After passing the preliminary homogeneity test, the RM candidates were dispensed into brown glass bottles, sealed with PTFE gasketed caps and stored at −20 °C. A total of 600 bottles of RM candidates were finally prepared, and each bottle contained 5 g of fish muscle powder.

To prevent sample contamination, the possible migration of target bisphenols from packaging bottles and their caps was investigated by substitute tests using methanol as the test media. No target bisphenols were detected after 25 days of soaking at room temperature. During the packaging process, the air inside the glass bottles was removed with nitrogen gas before screwing the caps, to maintain the stability of the candidates.

### 2.3. Preparation of Standard Solutions for Sample Analysis

A cocktail internal standard solution containing ^13^C_12_-BPA (25 ng/mL), ^13^C_12_-BPF (12.5 ng/mL), ^13^C_12_-BPS (25 ng/mL) and ^13^C_12_-BPAF (12.5 ng/mL) was prepared by mixing individual stock solutions with 50% methanolic water. The concentrations of the internal standards in this solution were selected according to the levels of the target bisphenols in the RM so that spiking 200 μL of this solution into 0.5 g of fish powder would reach isotope ratios of approximately 1:1. Individual intermediate solutions and working solutions of the four bisphenols were prepared gravimetrically by serial dilution of the stock standard solutions. For the quantification of the target analytes, a single-point calibration solution was prepared by mixing 0.0485 g of BPA working solution (0.0943 μg/g), 0.0392 g of BPF working solution (0.1034 μg/g), 0.0579 g of BPS working solution (0.0939 μg/g), 0.0287 g of BPAF working solution (0.0917 μg/g) and 200 μL of the cocktail internal standard solution, and diluting to 1 mL with 50% methanolic water.

### 2.4. Analytical Methods

An isotope dilution-liquid chromatography tandem mass spectrometry (ID-LC-MS/MS) method was employed in the homogeneity, stability and value assignment studies. This method was modified from our previous method for the detection of seven bisphenols in reed and Callitrichaceae [17]. The detailed steps were as follows: 0.5 g of fish powder was accurately weighed into a 50 mL glass centrifuge tube, spiked with 200 μL of the cocktail internal standard solution and kept at room temperature for 30 min. Two milliliters of ultrapure water was added, and the mixture was mixed for 1 min using a vortex mixer. Afterward, the mixed sample was extracted with 5 mL of acetonitrile by ultrasonication for 15 min, and centrifuged at 1740× *g* (3000 rpm) at 4 °C for 10 min to obtain the supernatant. Two milliliters of the supernatant was then transferred to another clean glass tube, 8 mL of water containing 0.3% formic acid was added, and the mixture was mixed thoroughly by vortexing. The resulting solution was passed through a Supelclean ENVI-Carb SPE cartridge (500 mg, 6 mL, product no. 57094, Sigma-Aldrich, St. Louis, MO, USA) preconditioned with 18 mL of methanol and 6 mL of water for further purification. The cartridge was subsequently washed with 6 mL of methanol-water (50:50, *v*/*v*), and eluted with 6 mL of methanol–acetone (40:60, *v*/*v*). The eluate was collected in another vial and concentrated to near dryness under nitrogen at 40 °C. The residue was dissolved in 1 mL methanol-water (50:50, *v*/*v*) for LC-MS/MS analysis.

Sample analysis was performed on an LCMS-8060 Liquid Chromatography Tandem Mass Spectrometer equipped with an electrospray ion source (Shimadzu, Kyoto, Japan). Liquid chromatographic separation was achieved by an Acquity UPLC BEH C18 column (2.1 mm × 100 mm, 1.7 μm) at 40 °C. The flow rate was set at 0.3 mL/min, and an injection volume of 5 μL was used. The mobile phase consisted of methanol (eluent A) and water (eluent B) with the gradient program set as follows: 0–1.0 min, 40% A; 1.0–6.0 min, 40–100% A; 6.0–7.0 min, 100% A; 7.0–7.1 min, 100–40% A; 7.1–9.5 min, 40% A. The mass spectrometer was operated in negative ion mode with the following parameters: interface voltage, 1.5 kV; Cone voltage, 30 V; ion source temperature, 300 °C; desolvation temperature, 250 °C; desolvation gas flow rate, 120 L/h; multiple reaction monitoring (MRM) mode. The detailed MRM parameters for each analyte are listed in Table 1. Data acquisition and processing were carried out using LabSolutions LCMS software version 5.99 (Shimadzu, Kyoto, Japan). Chromatographic peak integration was performed automatically based on peak-area quantification with a signal-to-noise (*S/N*) threshold of 3, and manual peak adjustment was applied where necessary. For quantification, the absolute mass (ng) of each target bisphenol in the sample injection was determined using the single-point calibration solution described in Section 2.3. The concentration of each analyte in the original fish sample (µg/kg) was then calculated by dividing this absolute mass by the test portion mass. The detailed calculation formula for single-point quantification is provided in Text S2.

### 2.5. Homogeneity Studies

To assess the homogeneity of the candidate RM, sixteen units were randomly selected from the 600 packages of fish muscle powder. Each selected unit was assigned a unique random number ranging from 1 to 16, and measured in triplicate for assessment of within-unit variability. In accordance with the requirements indicated in ISO 33405:2024 [25], units were measured in a completely random order to allow distinction between an analytical drift and a possible trend in the filling sequence. One-way analysis of variance (ANOVA) was used to quantify the within-unit standard deviation and between-unit standard deviation. The *F*-test was performed to assess the homogeneity of the candidate RM, comparing the *F* calculated value to the *F* critical value with the significance level α set to 0.05. All calculations were performed in Microsoft Excel 2019 (Microsoft Corporation, Redmond, WA, USA).

### 2.6. Stability Monitoring

To assess the short-term and long-term stability of the candidate RM, thirty-three units were randomly chosen, and divided into eleven groups with 3 units in each group. Six groups were stored at 60 °C for 0, 1, 2, 4, 7 and 10 days, respectively, to simulate transportation under stress conditions for short-term stability. The remaining five groups were stored at −20 °C for 0, 1, 3, 6, 12 months, respectively, to simulate typical storage conditions at the users’ sites for long-term stability. At the end of each predetermined time, one group was randomly removed from the designated temperatures and measured in duplicate. The results of the stability monitoring were assessed by linear regression analysis as a function of time. The slopes of the fitted lines were tested for significance using *t*-test to determine whether the analyte concentrations were stable. All calculations were performed in Microsoft Excel 2019 (Microsoft Corporation, Redmond, WA, USA).

### 2.7. Value Assignment

To determine the indicative values of the target bisphenols in the candidate RM, seven authoritative domestic laboratories with extensive experience in the analysis of bisphenols in food were invited to conduct a collaborative value assignment. Two units of the candidate RM were randomly distributed to each participating laboratory, and each unit was measured at least four times, using the ID-LC-MS/MS method described above. To ensure the reliability and traceability of the multi-laboratory data, standard solutions and spiked quality control samples, alongside a standard operating procedure of the analytical method, were provided to every participating laboratory. The measurement results from all collaborative laboratories were statistically tested using the Dixon, Grubbs, Cochran and D’Agostino tests by Microsoft Excel. The mean of all the results that passed the statistical tests was used as the assigned indicative value for each analyte. All calculations were performed in Microsoft Excel 2019 (Microsoft Corporation, Redmond, WA, USA).

## 3. Results and Discussion

### 3.1. Selection of Acquisition Methods for Incurred Materials

Conventional preparation of food reference materials typically relies on contaminant screening to identify incurred specimens that contain the target analytes at desirable concentrations [28,29,30]. This preliminary screening process, however, is often labor-intensive and yields raw materials with poor batch-to-batch reproducibility in concentration values. An alternative approach involves spiking standards into blank matrices [31,32], but the binding states between target compounds and the matrix in such artificially fortified materials may not adequately mirror those in naturally contaminated samples. Moreover, this strategy precludes further investigation of metabolite residues in the samples. In recent years, in vivo animal exposure has emerged as a viable strategy for producing raw materials for chemical contaminants in animal-derived foods [33,34]. By optimizing critical parameters, such as administration dosage and euthanasia timing, raw matrix samples containing target analytes at predefined concentrations can be reliably obtained. This approach not only yields matrix samples with authentic contamination profiles but also ensures consistent sample yield and concentration reproducibility. In our preliminary work, we monitored bisphenol concentrations in fish collected from supermarkets and various river basins. The measured levels of four target bisphenol analogues varied markedly among individual fish, rendering it impractical to collect a sufficient number of positive specimens with the desired concentrations through field screening alone. Consequently, we opted to prepare raw fish samples via in vivo exposure experiments.

Three exposure routes have been commonly employed for chemical administration in fish, including medicated bathing, oral feeding, and intraperitoneal injection [35]. Medicated bathing necessitates large volumes of aqueous solutions containing the target compounds, leading to substantial consumption of reference standards and potentially high inter-individual variability in exposure doses. Oral feeding, while more closely mimicking natural dietary intake, requires the additional preparation of compound-spiked feed. Moreover, due to hepatic first-pass metabolism, bisphenols display low bioavailability following gastrointestinal absorption in fish [36]. Intraperitoneal injection, although deviating slightly from natural environmental exposure pathways, enables convenient and precise control over individual dosing. Following injection, target compounds are rapidly absorbed into the systemic circulation through abdominal capillaries. On balance, this route offers superior performance with respect to operational feasibility, cost-effectiveness, and environmental friendliness. Accordingly, intraperitoneal injection was selected as the administration route in the present study.

### 3.2. Optimization of Preparation Conditions for Reference Material Candidates

In this study, the intraperitoneal injection dose and exposure time for tilapia were two important factors affecting the ultimate concentrations of bisphenol analogues in the RM candidates. We performed preliminary studies to determine the impact of various injection dosages and exposure times on the residual concentrations of target analytes in fish muscle powder (Table S1). The results indicated that when the injection doses of BPA, BPF, BPS and BPAF were set at 250 μg/kg, 500 μg/kg, 100 μg/kg and 50 μg/kg, respectively (Dose one), the concentrations of BPA, BPS and BPAF in fish muscle powder fluctuated only slightly between 3 h and 12 h after a single injection, whereas the concentration of BPF decreased significantly and was markedly lower than the levels of the other three analytes at 12 h. Adjusting injection doses of BPA, BPF, BPS, and BPAF to 100 μg/kg, 1500 μg/kg, 100 μg/kg, and 25 μg/kg, respectively (Dose two), resulted in more comparable average concentrations of the four target analytes in fish muscle powder after 12 h exposure, ranging from 33.8 to 90.7 μg/kg. To give enough time for muscle tissue collection following intraperitoneal injection, we ultimately decided to euthanize the tilapia at the 12 h mark, which also allowed for better synchronization of the experimental procedures across days. For instance, muscle samples can be taken at 7:00 a.m. the next day after an intraperitoneal injection is carried out at 7:00 p.m. the day before. Accordingly, the Dose two resulted in a more balanced concentration profile of the target analytes in the tissue after 12 h exposure and was therefore selected for the subsequent formal experiments.

In order to produce large batches of contaminated fish muscle powder, highly contaminated materials obtained from exposed tilapia needed to be diluted with blank materials to the desired target level. Before dilution, the prepared post-exposure fish muscle samples and non-exposure fish muscle samples were separately processed into muscle paste by cutting and homogenization, and the concentrations of target analytes in the homogenized samples were measured. The results showed that the concentrations of BPA, BPF, BPS and BPAF in the exposed fish muscle samples were 15.1 μg/kg, 14.3 μg/kg, 18.0 μg/kg and 10.2 μg/kg, respectively, while the concentrations of the four compounds in the unexposed fish muscle samples were 0.37 μg/kg, < LOD, 0.24 μg/kg and 0.08 μg/kg, respectively. Based on the results presented above, we decided to mix the exposed and unexposed fish muscle samples at a 1:9 mass ratio to prepare matrix RM candidates with the desired concentration levels. After mixing at this ratio, the concentrations of the four target analytes in the fish muscle are expected to be in the range of 1–2 μg/kg, comparable with the actual detection levels of these compounds in fish products reported in the literature [13,37,38]. Assuming an 80% freeze-drying dehydration rate, the concentrations of the four target analytes in the fish muscle powder were estimated to be approximately 5–10 μg/kg.

### 3.3. Optimization and Validation of the Sample Pretreatment Method

**Figure 1:**
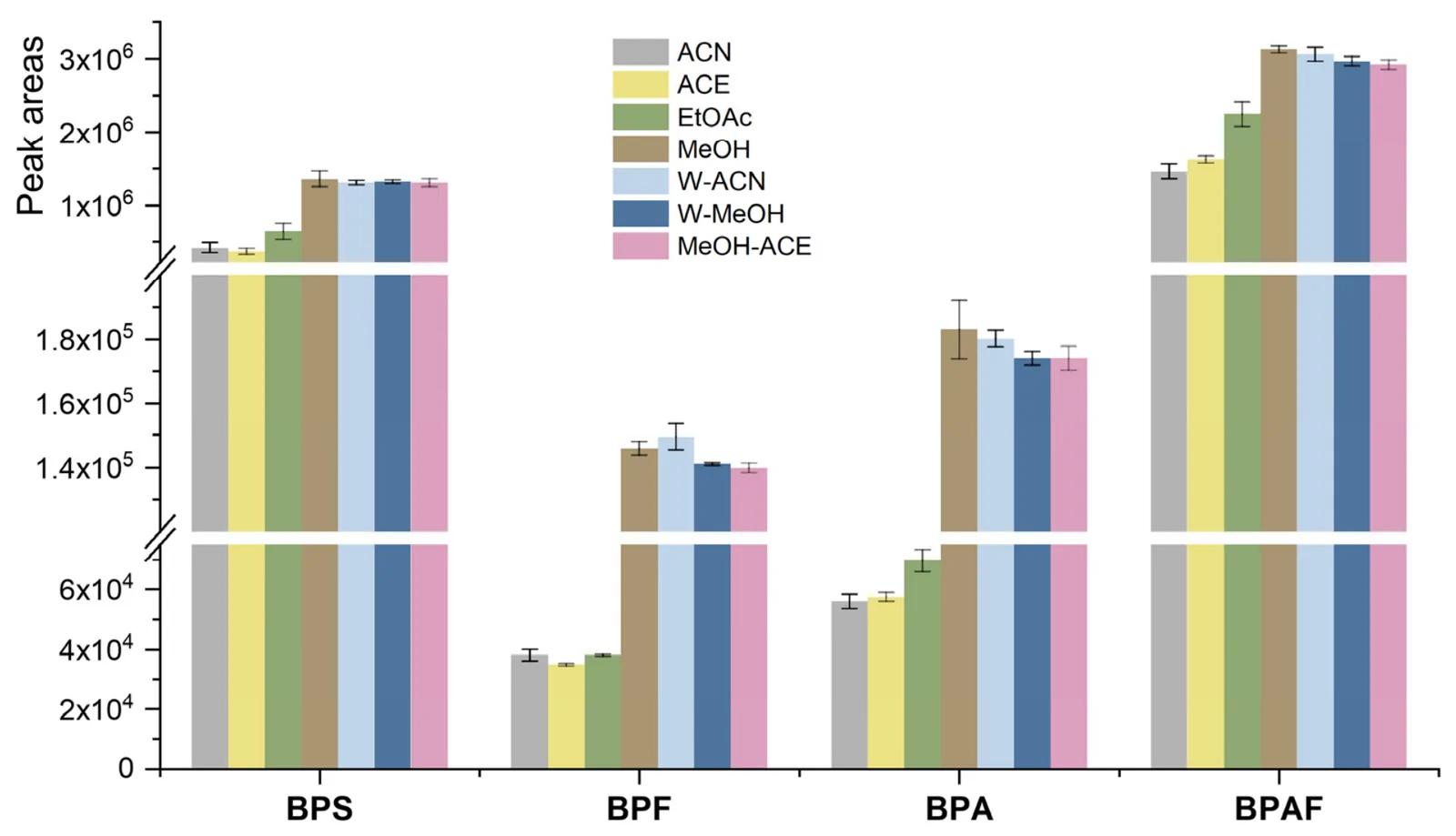
Extraction efficiencies of the four bisphenol analytes from positive fish muscle powder using seven different solvents, including acetonitrile (ACN), acetone (ACE), ethyl acetate (EtOAc), methanol (MeOH), water–acetonitrile (W-ACN), water–methanol (W-MeOH) and methanol–acetone (MeOH-ACE). The error bars represent the standard deviations (*n* = 3).

According to literature studies, acetonitrile was usually selected as the extraction solvent for bisphenols in high-moisture foods [18,20,21]. For dried samples, it is generally necessary to re-dissolve them with an appropriate amount of water before extraction with an organic solvent [18]. However, some studies have also directly used organic solvents to extract dried samples [19,39,40], which may lead to suboptimal recoveries unidentifiable by spike tests. To select a suitable extraction solvent for our fish muscle powder, we evaluated and compared the extraction efficiencies of several solvent systems, including 5 mL acetonitrile, 5 mL acetone, 5 mL ethyl acetate, 5 mL methanol, water–acetonitrile (re-dissolved in 2 mL water and extracted with 5 mL acetonitrile), water–methanol (re-dissolved in 2 mL water and extracted with 5 mL methanol) and 5 mL methanol–acetone (1:1, *v*/*v*), using positive powder samples obtained by fish administration. The results shown in Figure 1 indicate that methanol yielded higher extraction efficiency for the four bisphenol analogues in fish muscle powder relative to other neat organic solvents applied without water addition. The extraction efficiencies of methanol, water–acetonitrile, water–methanol and methanol–acetone (1:1, *v*/*v*) were essentially equivalent. However, when water–acetonitrile was used as the extraction solvent, the resulting sediment after centrifugation was more compact and less prone to forming flocculent floaters, which facilitated the transfer of the supernatant. Thus, the final extraction procedure involved reconstituting the fish muscle powder in water followed by acetonitrile extraction. The volume of the extraction solvent was also optimized by comparing the peak areas of the target compounds obtained with 5 mL of acetonitrile versus 10 mL of acetonitrile. Similar peak-area responses were obtained for the two solvent volumes, and 5 mL of acetonitrile was utilized in the determinations.

After solvent extraction and low-temperature centrifugation at 4 °C, an additional clean-up procedure was performed using graphitized carbon black (GCB) SPE cartridges, namely, Supelclean ENVI^TM^-Carb, to reduce the remaining interferences in the supernatant. The GCB cartridges were washed with 6 mL methanol three times before sample loading to eliminate possible background contamination. The pH values of the loading solution were adjusted to approximately 3.5 with formic acid to reduce the ion-exchange interaction between the analytes and GCB sorbents, since some acidic bisphenols, e.g., BPS, tend to strongly bond with the GCB sorbents by anion exchange and were difficult to be eluted off [41]. The purification efficiencies of the GCB cartridges were evaluated by the matrix effect (ME) based on the following formula: ME = (1 − S_m_/S_0_) × 100%, where S_m_ is the slope of the matrix-matched standard curve of each analyte, and S_0_ is the slope of the neat standard curve prepared with pure solvent at the same concentrations. An ME value of 0% represents no matrix effect, whereas ME > 0% and ME < 0% represent signal suppression and enhancement, respectively. Fish muscle powder samples devoid of the four bisphenols were selected, extracted, purified and dried according to the pretreatment method. The matrix-matched standard solutions were prepared by reconstituting the residue with 1 mL of neat standard solution. The results presented in Table 2 indicate that the ME of every analyte was less than 21%, demonstrating that GCB cartridges were effective in the purification of fish samples and that the pretreatment method exhibited only a slight signal suppression effect. We further evaluated the compensatory role of isotopic internal standards in mitigating matrix interference by calculating ME with the slopes of isotope dilution calibration curves. As shown in Table 2, the ME values corrected via isotope dilution are all less than 4%, indicating neither significant suppression nor enhancement of the target analytes. This finding suggests that the ME impact on accurate quantification can be ignored after isotopic internal standard correction.

To ensure the accuracy of our analytical method, a recovery experiment was performed by analyzing blank fish powder samples spiked with four analytes at concentrations of 5 and 10 μg/kg, which are close to the initial measured levels in our RM candidates. Six replicates were prepared for each spiking concentration to determine the method precision, expressed as the percent relative standard deviations (RSDs) of the recoveries. In addition, a seven-point calibration curve was created for each analyte by plotting the peak area ratios of the analyte to the internal standard versus the mass of the analyte to assess the linearity of the quantitative method (Text S2). Both back-calculation and residual analysis were conducted to fully validate the calibration performance. The limits of detection (LODs) and limits of quantification (LOQs) for the four analytes were also investigated, and defined as the lowest spiked concentration in blank fish powder samples that can yield a *S/N* ratio greater than 3 and a *S/N* ratio greater than 10, respectively. As summarized in Table 2, the mean recoveries of the analytes ranged from 95.3 to 106.3%, with RSDs at every spiking level less than 7.0%, demonstrating good accuracy and precision of the method for complex fish matrices. Good linearity was observed for the calibration curves of all analytes, with all correlation coefficients (*R*^2^) greater than 0.9986. All calibration points of the calibration curves exhibited back-calculated biases within ±7%, well below the ±15% acceptance criterion (Table S2). Residual plots showed random distribution of residuals around zero without obvious curvature or heteroscedasticity, confirming the excellent fitness of the linear model (Figure S1). All intercepts of the calibration curves were not significantly different from zero by *t*-test (*p* > 0.05). These results suggest that the single-point calibration method can be applied for the quantitative analysis of the target analytes in RM candidates. The LODs and LOQs for BPA and BPF in fish muscle powder were determined to be 0.15 μg/kg and 0.50 μg/kg, respectively. For BPS and BPAF, the corresponding LODs and LOQs were 0.015 μg/kg and 0.050 μg/kg, respectively. The sensitivity of this method was adequate for the concentration levels of the four bisphenol analogues present in our RM candidates.

Given the well-documented background contamination issues in bisphenol analogues analysis, special quality control measures were taken during our analytical procedure, which was crucial for ensuring data accuracy and reliability. All glassware in our experiment was washed with ethanol, rinsed with Milli-Q water, and baked for 4 h at 400 °C before use. Methanol, acetonitrile and water from different manufacturers and brands were screened, and products free of target analytes were selected as extraction solvents, SPE eluents and LC mobile phases. A procedural blank and a blank matrix extract were applied to every ten test samples to evaluate background contamination. The peaks of the target analytes in these blanks were controlled below the detection limit of the method.

### 3.4. Homogeneity Assessment of RM Candidates

**Figure 2:**
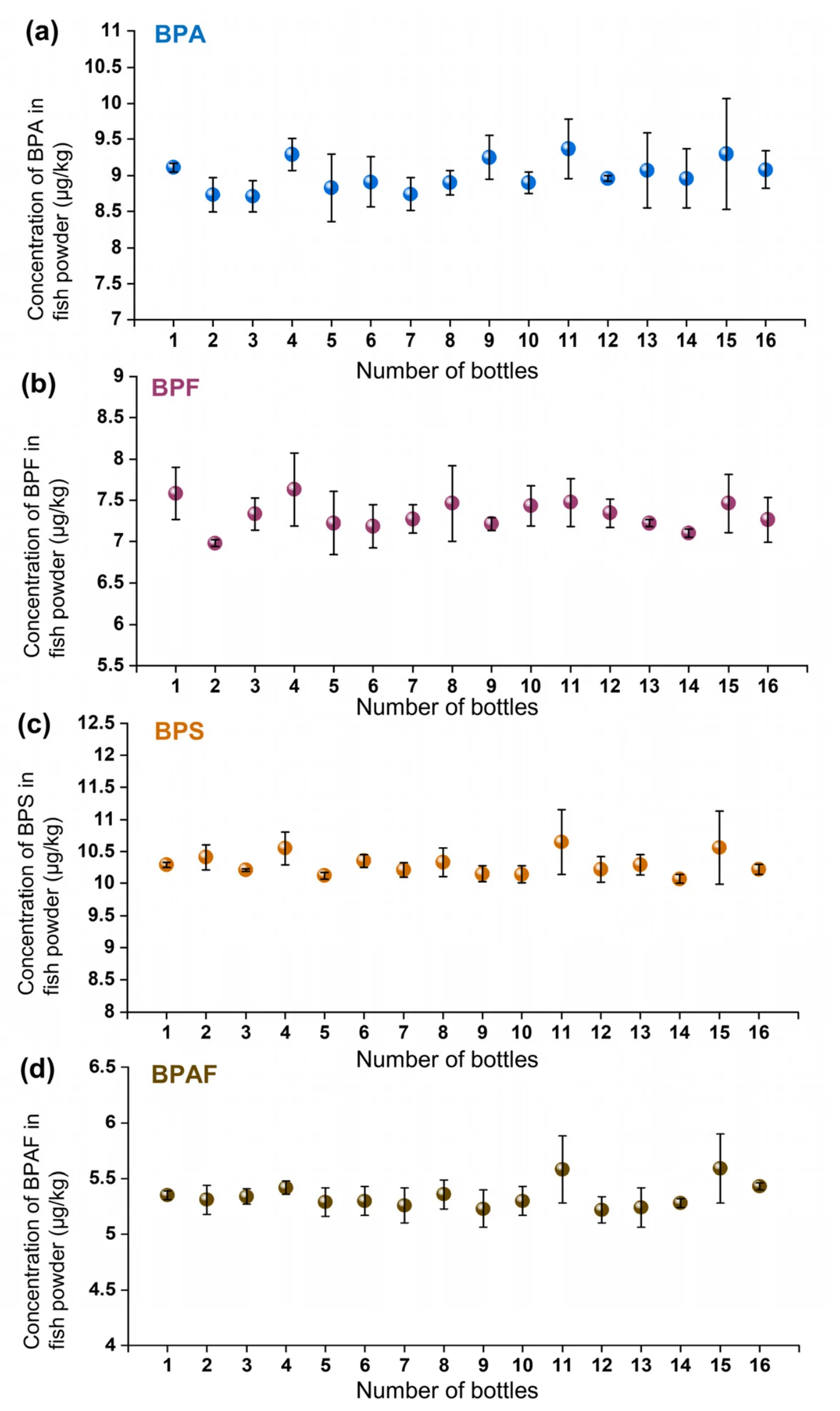
Homogeneity assessment results of (**a**) BPA, (**b**) BPF, (**c**) BPS and (**d**) BPAF in the fish muscle powder RM. The error bars represent the standard deviations obtained from three independently weighed and fully processed test portions prepared from each selected bottle (*n* = 3).

The homogeneity of the candidate RM was evaluated to determine whether the amounts of the four target analytes were equally distributed among different bottles, according to ISO 33405:2024 [25]. The detection data obtained using our ID-LC-MS/MS method (Tables S3–S6) were subjected to ANOVA to assess the variation between and within samples. Table 3 summarizes the results of the ANOVA, and Figure 2 graphically presents the concentration values of the four target analytes from the sixteen randomized RM samples. The *F*-value for the BPS was equal to 1.569, which was lower than the critical *F*-value (1.992) at the 95% confidence level (α = 0.05). Similarly, the *F*-values for BPA, BPF and BPAF were all less than the critical *F*-value. These results indicate no statistically significant differences in analyte concentrations among samples, suggesting that the RM was sufficiently homogeneous at the 95% confidence level.

The uncertainty introduced by potential heterogeneity between bottles was estimated according to the two formulas as follows.

(1) $$u_{\mathrm{bb}}\;=\sqrt{\frac{{MS}_{\mathrm{between}}-{MS}_{\mathrm{within}}}{n}}$$

(2) $$u_{\mathrm{bb}}^{*}\;=\sqrt{\frac{{MS}_{\mathrm{within}}}{n}}\cdot \sqrt[{4}]{\frac{2}{N-m}}$$

where *u_bb_* denotes the uncertainty value of homogeneity for *MS*_between_ > *MS*_within_. $u_{\mathrm{bb}}^{*}$ denotes the uncertainty value of homogeneity for *MS*_between_ < *MS*_within_*. MS*_between_ and *MS*_within_ refer to the mean squares between and within bottles, respectively. *n*, *N* and *m* denote the number of replicate measurements per subsample (set as 3), number of total detected samples for homogeneity study (set as 48) and number of units (set as 16), respectively. The larger value between *u*_bb_ and $u_{\mathrm{bb}}^{*}$ was used as the uncertainty value of homogeneity.

Furthermore, the relative standard deviation of *u*_bb_, represented by *u*_bb,rel_, was calculated based on Formula (3).

(3) $$u_{\mathrm{bb},\mathrm{rel}}=\frac{u_{\mathrm{bb}}}{\bar{x}}$$

where $\bar{x}$ represents the average of all the results of the homogeneity study for each analyte. The calculated *u*_bb,rel_ values of the four analytes ranged from 0.99% to 1.24%.

### 3.5. Stability Studies of RM Candidates

Stability reflects the ability of an RM to maintain its characteristic values under defined storage conditions over a specified time. It is a crucial parameter for evaluating the accuracy and reliability of an RM during its designed shelf life. In this study, the short-term and long-term stability of our RM were both investigated at different experimental temperatures and times. The former was conducted at 60 °C for 10 days to establish appropriate transportation condition, whereas the latter one was conducted at −20 °C for 12 months to assess potential analyte degradation in the candidate RM during storage. The mass fraction concentrations of all samples at each experimental temperature (Tables S7 and S8) were statistically analyzed using linear regression analysis by fitting a straight line between time and concentrations. The slope of the fitted straight line (*β*_1_) for each analyte was calculated, and its significance was assessed by the *t* test, which compared the result of *β*_1_*/S_β_*_1_ to the critical *t*-value (*t_(0.95,n−2)_*), where *S_β_*_1_ and *n* represent the standard error of the slope and the monitoring time points, respectively.

**Figure 3:**
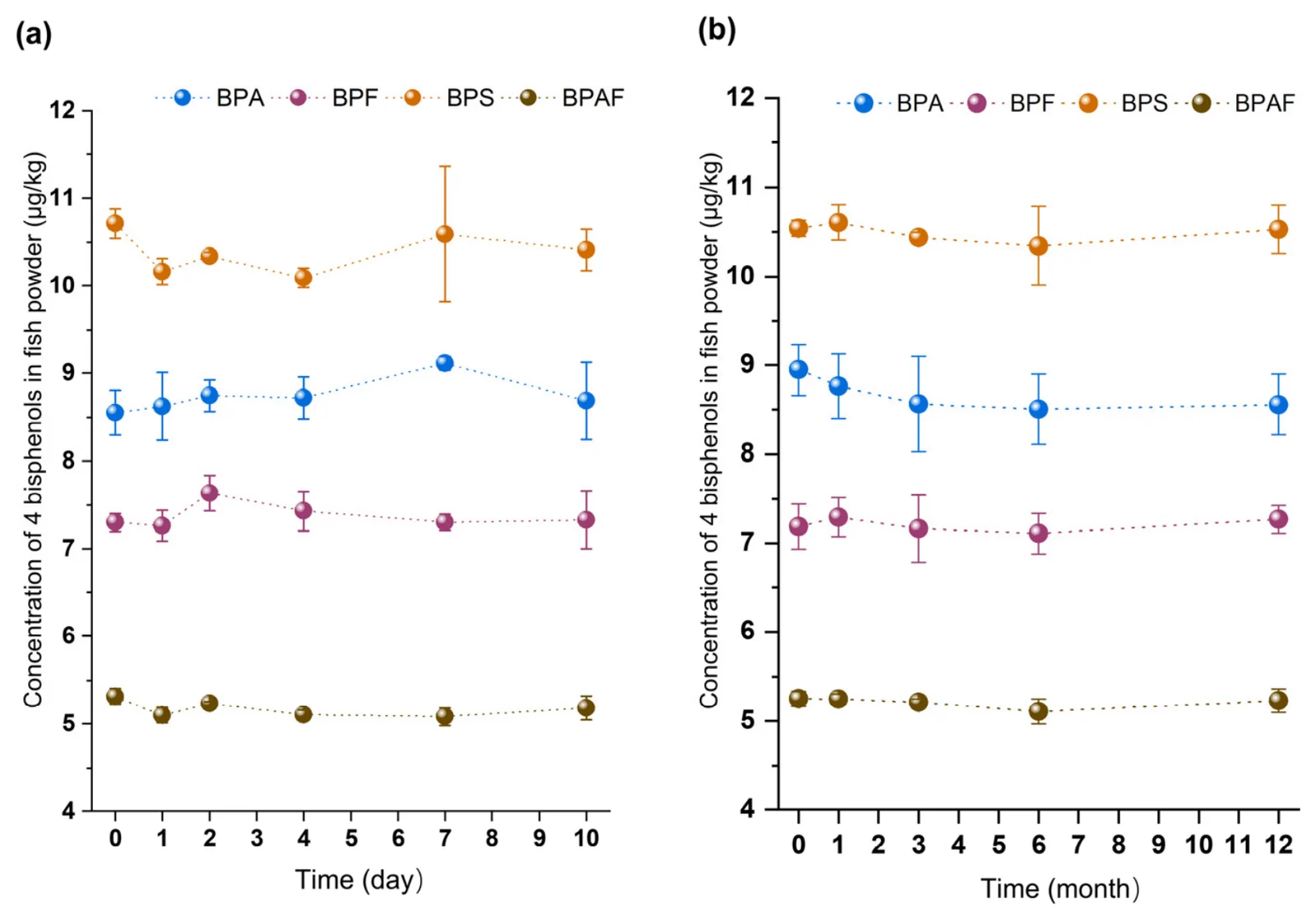
Assessment results of (**a**) short-term stability at 60 °C and (**b**) long-term stability at −20 °C for four bisphenol analytes in the fish muscle powder RM. Each plotted value is the overall mean of three independent RM units, while duplicate measurements within each unit were averaged first. The error bars represent the standard deviations across the three units (*n* = 3) for every monitoring point.

As shown in the trend plots in Figure 3, no apparent increase or decline could be visually observed for the analyte concentrations during both short-term and long-term experiments. Table 4 summarizes the data calculated from the linear regression analysis. For all target analytes, the values of *β*_1_*/S_β_*_1_ were less than *t_(0.95,n−2)_* at a 95% confidence level (*α* = 0.05) and *n* − 2 freedom degrees (4 and 3 for short-term and long-term stability, respectively). These results indicate that the slopes of the fitted straight lines were not statistically significant, suggesting that the four bisphenol analogues in our RM remained stable for 10 days at 60 °C or for 12 months at −20 °C. It can be concluded that this RM can be transported at ambient temperature for at least 10 days without special temperature control measures, which meets the demand of delivery to laboratories worldwide.

The relative uncertainties of short-term stability (*u*_sts,rel_) and long-term stability (*u*_lts,rel_) were evaluated according to the following formulas:

(4) $$u_{\mathrm{sts}}\;\mathrm{or}{\;u}_{\mathrm{lts}}\;=\;S_{\beta 1}\cdot \;t$$

(5) $$u_{\mathrm{sts},\mathrm{rel}}=\frac{u_{\mathrm{sts}}}{\bar{x}}$$

(6) $$u_{\mathrm{lts},\mathrm{rel}}=\frac{u_{lts}}{\bar{x}}$$

where *u*_sts_ and *u*_lts_ represent the uncertainties of short-term stability and long-term stability, respectively, *S_β_*_1_ is the standard deviation of the stability slope, *t* is the monitoring time of stability, and $\bar{x}$ is the average of all concentrations in the stability study.

### 3.6. Characterization of RM Candidates

**Figure 4:**
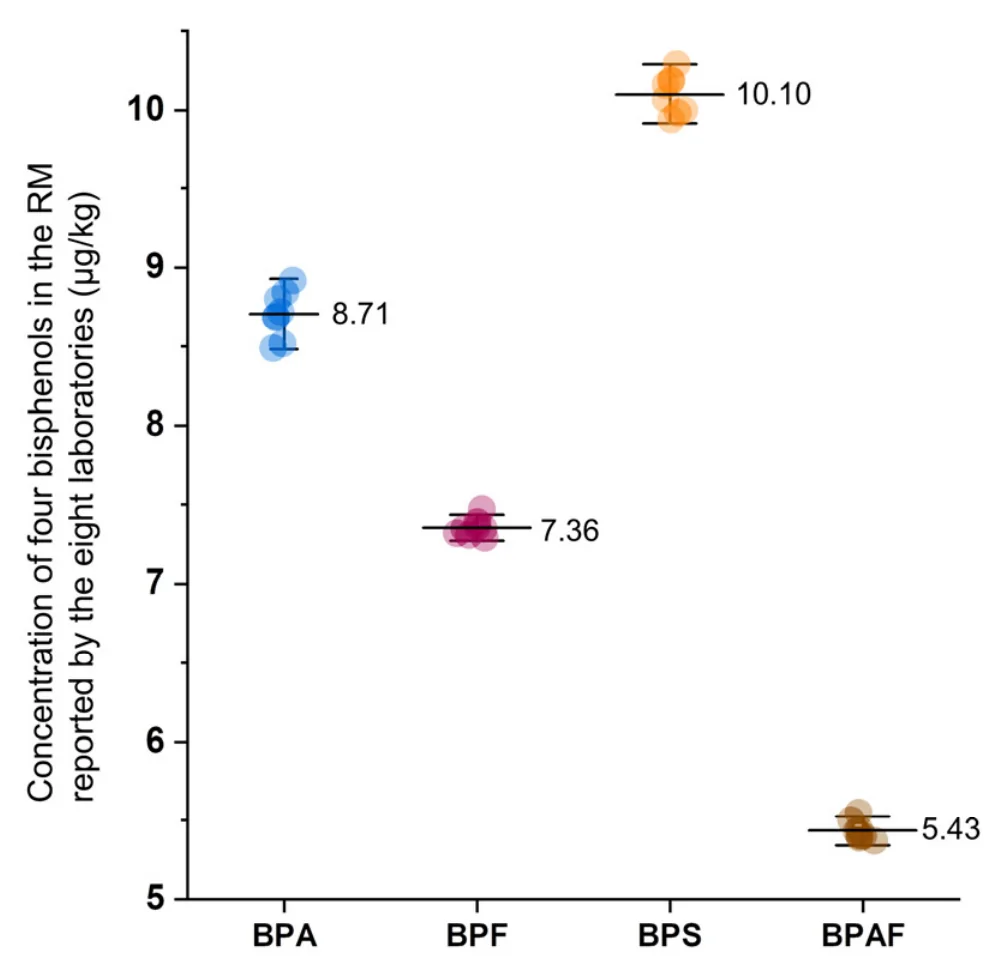
Concentrations of four bisphenol analytes reported by the eight laboratories involved in the characterization process of the fish muscle powder RM. Each plotted point denotes the value reported by an individual laboratory. The solid lines within the plotted points represent the mean concentrations of all eight laboratories, with the error bars indicating the between-laboratory standard deviations (*n* = 8).

Eight authoritative domestic laboratories, with extensive experience in relevant analytical fields, were involved in the characterization process of RM candidates. Each laboratory conducted four measurements per sample, resulting in eight data points per laboratory. The reliability of the data submitted by every laboratory was first evaluated according to the blank and spiked quality control samples, and no data were technically excluded. The accepted data were then statistically analyzed using the Dixon’s and Grubbs’ tests to check for outliers within each laboratory. The Cochran test was used to evaluate the agreement of the data among different laboratories. At a confidence level of 95%, no outliers were identified for the four target bisphenols by these tests. Therefore, all detection data from eight laboratories were adopted for value assignment (Figure 4, Tables S9–S12). The D’Agostino test did not detect a statistically significant departure from normality for these data at a significant level of α = 0.05. However, it should be noted that this normality test has limited statistical power, because only eight laboratory-derived values were included in the evaluation. The mean values of these data were calculated and considered as the appropriate estimates for the indicative values of the target analytes in our RM (Table 5).

The uncertainty introduced during the value assignment process involves two main components: type A uncertainty (*u*_A_) from the collaborative certification of multiple laboratories, and type B uncertainty (*u*_B_) from the sample measurement processes. The type A uncertainty was calculated using Formula (7) and (8) as follows:

(7) $$u_{A}\;=\sqrt{\frac{{\sum_{i\;=\;1}^{m}\left({\bar{x}}_{i}-\overset{̿}{x}\right)}^{2}}{m\;\times \;\left(m-1\right)}}$$

(8) $$u_{A,\mathrm{rel}}=\frac{u_{A}}{\overset{̿}{x}}$$

where $\bar{x_{i}}$ is the mean of data from each laboratory, $\overset{̿}{x}$ is the overall mean from all laboratories, *m* is the number of the participating laboratories and *u*_A,rel_ is the relative uncertainty of type A.

The potential sources of type B uncertainty were systematically evaluated according to the quantitative method in this study. The relative combined uncertainty of type B (*u_B,rel_*) was estimated based on Formula (9):

(9) $$u_{B,rel}\;=\;\sqrt{{u_{1,\mathrm{rel}}}^{2}\;+\;{u_{2,\mathrm{rel}}}^{2}\;+\;{u_{3,\mathrm{rel}}}^{2}\;+\;{u_{4,\mathrm{rel}}}^{2}\;+{{\;u}_{5,\mathrm{rel}}}^{2}}$$

where *u*_1,rel_ and *u*_2,rel_ denote the relative uncertainties introduced during the preparation of intermediate solutions and working solutions, respectively; *u*_3,rel_ denotes the relative uncertainty introduced during the preparation of a single-point calibration solution; *u*_4,rel_ denotes the relative uncertainty introduced by the mass of the internal standard added into the calibration solution and test samples; and *u*_5,rel_ denotes the relative uncertainty introduced during sample weighing.

### 3.7. Uncertainty Assessment

The sources of uncertainty for the characteristic values of our RM consist of three components, including characterization (*u*_A_ and *u*_B_), homogeneity (*u*_bb_) and stability (*u*_sts_ and *u*_lts_). To estimate the relative combined uncertainty of the candidate RM (*u*_RM,rel_), these components were combined based on Formula (10):

(10) $$u_{\mathrm{RM},\mathrm{rel}}\;=\;\sqrt{{u_{A,\mathrm{rel}}}^{2}\;+{{\;u}_{B,\mathrm{rel}}}^{2}\;+\;{u_{\mathrm{bb},\mathrm{rel}}}^{2}\;+\;{u_{\mathrm{lts},\mathrm{rel}}}^{2}\;+{{\;u}_{\mathrm{sts},\mathrm{rel}}}^{2}}$$

The relative expanded uncertainty (*U*_RM,rel_) was then calculated by multiplying the *u*_RM,rel_ with a coverage factor of *k* = 2 at a confidence level of 95%, and the standard expanded uncertainty (*U*_RM_) was calculated by multiplying the *U*_RM,rel_ with the RM’s characteristic value. Table 5 summarized the uncertainty sources and evaluated results. The expanded uncertainties (absolute value) ranging from 0.42 to 0.80 μg/kg were observed for the four bisphenols in the fish muscle powder. These relatively low-magnitude uncertainty estimates may be attributed to several experimental factors. First, ID-LC-MS/MS was employed as the core characterization method, which can offer high trueness and precision for quantification. Second, the inter-laboratory collaborative assignment may help reduce random measurement bias by aggregating replicate datasets generated by multiple independent laboratories. Third, as shown in Table 5, characterization-associated sample measurement processes and stability appear to represent the major contributions to the overall uncertainty budget, while the relative contribution from between-unit homogeneity (*u*_bb,rel_) is comparably small, which may be ascribed to the satisfactory homogeneity. Nevertheless, these uncertainty estimates are obtained within the current single-method analytical framework, so that potential systematic bias cannot be fully excluded.

Our uncertainty evaluation procedure complies with ISO 33405:2024 [25]. All mandatory uncertainty components for the assigned indicative values, namely type A and type B characterization contributions, between-unit homogeneity, as well as short-term and long-term stability, were identified, quantified and combined via the root-sum-of-squares approach. ISO 33405:2024 does not specify fixed numerical thresholds for expanded uncertainty values. Instead, the core requirement is that all relevant uncertainty sources should be comprehensively recognized and appropriately integrated into the final uncertainty budget, which has been satisfied in the present study.

### 3.8. Strengths and Limitations

Although widespread human exposure to bisphenol analogues have raised global concern, public reports on matrix RMs for these compounds remain very limited in the current literature. The U.S. National Institute of Standards and Technology (NIST) recently released a matrix RM for various organic contaminants in human urine (No. SRM 3672a), which included three bisphenol analogues (BPA, BPF and BPS) with non-certified concentration values of 6.70 ± 0.35 ng/g, 1.028 ± 0.054 ng/g and 1.111 ± 0.043 ng/g, respectively [42]. The authors of this study previously developed a matrix RM candidate for four bisphenol analogues in infant formula milk powder. This candidate, which has not yet been certified by multi-laboratory collaborative value assignment, contained BPA, BPF, BPS and BPAF at concentration levels of approximately 15.77–34.61 μg/kg [43]. Compared with these existing studies, to the best of our knowledge, the present research is the first to report a matrix RM for bisphenol analogues in fish muscle, filling the gap of bisphenol RMs suitable for meat matrices in the field of food safety. Additionally, after converted to wet weight based on a dehydration rate of 80%, the concentration levels (approximately 1.09 to 2.02 μg/kg) of the four bisphenol analogues in this RM are comparable to the actual detection levels in fish products reported in previous studies [13,37,38]. This makes our RM more practically valuable in applications such as analytical method validation and laboratory quality control.

Despite the aforementioned advantages, the present study also has certain limitations. First, our RM was specifically developed from a tilapia muscle matrix, and its commutability against other matrices has not been formally assessed. Without experimental evidence, its equivalence to authentic samples from alternative meat matrices, such as other fish species (e.g., salmon), shrimp, pork and beef, cannot be confirmed. Given that different matrix compositions and interferences may significantly influence extraction recovery, further validation across these matrices is urgently required in subsequent work. Second, the long-term stability of the RM at room temperature (25 °C) and refrigerated temperature (4 °C) are not yet available, and further stability investigations are needed to provide a more comprehensive data basis for the long-term storage of our RM. Third, the current RM only includes four bisphenol analogues, and the number of target compounds needs to be expanded to cover more bisphenol analogues that are widely present in food and pose potential health risks. Moreover, it should be noted that the present RM may not be well suited for the quantification of bisphenols at very low trace levels, owing to its current concentration range. Considering the growing concern over health effects associated with low-level bisphenol exposure, future studies will focus on developing RMs with lower concentration levels to better meet the needs of trace bisphenol detection in food safety monitoring. Finally, it is also worth noting that the value assignment in this study relied on a single analytical platform (ID-LC-MS/MS) through an inter-laboratory collaborative exercise. Although this approach is recognized in relevant ISO standards, the use of a single measurement method may still increase the risk of systematic bias. Future work will therefore incorporate orthogonal techniques such as LC-HRMS and/or GC-MS to establish a more robust certification strategy.

## 4. Conclusions

To the best of our knowledge, this study presented the first development of a matrix RM for the determination of four bisphenols in fish muscle, considering the scarcity of available RMs for bisphenols in the field of food safety. After the intraperitoneal administration of target compounds to tilapia, approximately 3 kg of candidate fish muscle powder was processed by means of mincing, homogenizing, freeze-drying, crushing, milling, three-dimensional mixing and filling. Homogeneity assessment and stability monitoring of the powder was performed using an ID-LC-MS/MS method with good accuracy and precision. The RM exhibited satisfactory homogeneity, and could be stably stored for at least 10 days at 60 °C or for 12 months at −20 °C, supporting its reliability for the intended use. The assignment of indicative values of bisphenols and the evaluation of uncertainties were also conducted according to the requirements of ISO 33405:2024. The finally assigned indicative values and expanded uncertainties for the RM ranged from 5.43 to 10.10 μg/kg and 0.42 to 0.80 μg/kg (*k* = 2), respectively. This newly developed RM will be a valuable tool in analytical method validation and laboratory quality control for the accurate detection of bisphenols in fish muscle. Its promising application will help establish and maintain reliable monitoring results in fish-derived food products. Furthermore, by enabling accurate quantification of bisphenols in fish, this RM can also help provide technical support for dietary exposure calculation, thus strengthening the practical value of this research in bisphenol risk assessment.

## Figures and Tables

**Table 1:** Multiple reaction monitoring (MRM) parameters of the analytes in fish meat powder.

Compounds	Parent Ion (*m/z*)	Daughter Ion (*m/z*)	Collision Energy (eV)	Cone Voltage (V)
BPA	227.0	212.0 */133.0	18/25	30
BPF	199.1	93.0 */105.0	22/22	30
BPS	249.0	108.0 */156.0	21/15	30
BPAF	335.1	265.0 */197.0	22/37	30
^13^C_12_-BPA	239.1	224.1	19	30
^13^C_12_-BPF	211.1	99.1	23	30
^13^C_12_-BPS	261.1	114.1	26	30
^13^C_12_-BPAF	347.1	277.1	23	30

Note: * for quantitative ions.

**Table 2:** The accuracy, precision, linearity, sensitivity and matrix effect of the developed method for the determination of four bisphenols in fish muscle powder.

Compounds	Spiking Level (μg/kg)	Recovery (%)	RSD (%)	Linear Range (ng)	*R* ^2^	LOD (μg/kg)	LOQ (μg/kg)	ME_1_ (%)	ME_2_ (%)
BPA	5	98.2	2.2	0.87–6.54	0.9987	0.15	0.50	20.9	−3.5
	10	102.0	4.0						
BPF	5	105.8	4.6	0.97–7.27	0.9989	0.15	0.50	5.4	4.4
	10	96.5	3.5						
BPS	5	95.3	6.8	0.92–6.94	0.9999	0.015	0.050	5.8	1.6
	10	106.3	4.2						
BPAF	5	104.0	2.6	0.87–6.56	0.9992	0.015	0.050	5.0	2.5
	10	101.0	1.7						

Note: RSD is the relative standard deviation of recovery; linear ranges are expressed as absolute mass (ng) because calibration curves were constructed using analyte mass as the *x*-axis (Text S2); *R*^2^ is the correlation coefficient of calibration curve; LOD is the limit of detection; LOQ is the limit of quantification; ME_1_ is the matrix effect without isotope standard correction; ME_2_ is the matrix effect after isotope standard correction.

**Table 3:** ANOVA results and estimated uncertainty for homogeneity study of four bisphenols in candidate materials.

Compounds	Concentrations (μg/kg)	Mean Squares (MS, μg/kg)		*F*_value_	*F*_critical_ (α = 0.05)	*S*_bb_ (μg/kg)	*u*_bb_ (μg/kg)
		*MS*_between_ (*m* = 16)	*MS*_within_ (*n* = 3)				
BPA	9.01	0.1356	0.1225	1.107	1.992	0.066	0.101
BPF	7.32	0.0939	0.0733	1.281		0.083	0.083
BPS	10.30	0.0866	0.0552	1.569		0.102	0.102
BPAF	5.34	0.0371	0.0238	1.559		0.066	0.066

Note: *MS*_between_ is the mean squares between bottles; *MS*_with_ is the mean squares within bottles; *F*_value_ is the value of the *F* statistic in ANOVA; *F*_critical_ is the critical *F* value at a 95% confidence level; *S*_bb_ is the between-bottle standard deviation; *u*_bb_ is the final uncertainty value of homogeneity.

**Table 4:** Results of linear regression analysis for the evaluation of short-term and long-term stability.

Compounds	Short-Term Study (60 °C for 10 Days)				Long-Term Study (−20 °C for 12 Months)			
	*β*_1_ (μg/kg/Day)	*S_β_*_1_ (μg/kg/Day)	|*β*_1_/*S_β_*_1_|	*t*_critical_	*β*_1_ (μg/kg/Month)	*S_β_*_1_ (μg/kg/Month)	|*β*_1_/*S_β_*_1_|	*t*_critical_
BPA	0.0257	0.0222	1.158	2.776	−0.0270	0.0157	1.720	3.182
BPF	−0.0048	0.0177	0.271		0.0018	0.0089	0.202	
BPS	0.0031	0.0309	0.100		−0.0049	0.0118	0.415	
BPAF	−0.0093	0.0111	0.838		−0.0037	0.0067	0.552	

Note: *β*_1_ is the slope of the regression line for each analyte in stability studies; *S_β_*_1_ is the standard error of the slope; *β*_1_/*S_β_*_1_ represents the ratio of the regression line slope to the standard error of the slope, which serves as the test statistic for the *t*-test to evaluate the significance of the slope; *t*_critical_ is the critical *t*-value at a 95% confidence level (α = 0.05), with 4 and 3 degrees of freedom for the short-term and long-term stability studies, respectively.

**Table 5:** Assigned indicative values with uncertainties for the concentration of the four bisphenols in the RM.

Compounds	Assigned Indicative Values (μg/kg)	Sources of Relative Standard Uncertainty (%)					*u*_RM,rel_ (%)	*U*_RM,rel_ (*k* = 2) (%)	*U*_RM_ (*k* = 2) (μg/kg)
		*u*_A,rel_	*u*_B,rel_	*u*_bb,rel_	*u*_lts,rel_	*u*_sts,rel_			
BPA	8.71	0.60	1.95	1.12	2.17	2.54	4.07	8.14	0.71
BPF	7.36	0.26	2.19	1.13	1.48	2.41	3.76	7.53	0.56
BPS	10.10	0.43	1.86	0.99	1.36	2.97	3.91	7.82	0.80
BPAF	5.43	0.39	2.50	1.24	1.53	2.14	3.85	7.71	0.42

Note: *u*_A,rel_ is the type A relative uncertainty from the collaborative certification of multiple laboratories; *u*_B,rel_ is the type B relative uncertainty from the sample measurement process; *u*_bb,rel_ is the relative uncertainty from homogeneity assessment; *u*_lts,rel_ is the relative uncertainty from long-term stability study; *u*_sts,rel_ is the relative uncertainty from short-term stability study; *u*_RM,rel_ is the combined relative uncertainty of the RM; *U*_RM,rel_ is the expanded relative uncertainty of the RM; *U*_RM_ is the expanded absolute uncertainty of the RM.

## Data Availability

The original contributions presented in the study are included in the article/Supplementary Material; further inquiries can be directed to the corresponding authors.

## Supplementary Materials

The following supporting information can be downloaded at https://www.mdpi.com/article/10.3390/foods15172986/s1, Text S1: Detailed information for tilapia husbandry and exposure in this study; Text S2: Equations for single-point quantification, calibration curve fitting, homogeneity assessment, stability assessment and collaborative value assignment; Table S1: Concentrations of four analytes in fish muscle powder at different injection doses and exposure times (μg/kg, *n* = 3); Table S2: Linearity and back-calculation results for the calibration curves of four bisphenol analogues; Table S3: Homogeneity assessment results for BPA in the RM of fish muscle powder (μg/kg); Table S4: Homogeneity assessment results for BPF in the RM of fish muscle powder (μg/kg); Table S5: Homogeneity assessment results for BPS in the RM of fish muscle powder (μg/kg); Table S6: Homogeneity assessment results for BPAF in the RM of fish muscle powder (μg/kg); Table S7: Short-term stability test results of bisphenol analytes in the RM of fish muscle powder at 60 °C; Table S8: Long-term stability test results of bisphenol analytes in the RM of fish muscle powder at −20 °C; Table S9: BPA concentrations in the candidate RM determined by eight laboratories participating in collaborative value assignment (μg/kg); Table S10: BPF concentrations in the candidate RM determined by eight laboratories participating in collaborative value assignment (μg/kg); Table S11: BPS concentrations in the candidate RM determined by eight laboratories participating in collaborative value assignment (μg/kg); Table S12: BPAF concentrations in the candidate RM determined by eight laboratories participating in collaborative value assignment (μg/kg). Figure S1: Residual plots for the seven-point calibration curves of BPA, BPF, BPS and BPAF. Residual values are plotted against the standard mass (ng) of each analyte in calibration solutions.
