# Supplementary material for: Development of a Novel Matrix Reference Material for Four Bisphenols in Fish Muscle Powder
Source: Foods. 2026 Aug 25;15(17):2986. doi: 10.3390/foods15172986 (PMC13564519; doi:10.3390/foods15172986)
Supplement: Supplementary file 1 [file foods-15-02986-s001.zip › foods-4475096-supplementary.pdf]

Supplementary materials

Development of a novel matrix reference material for four bisphenols in fish muscle powder

Yunjia Yang [1], Xiao Xu [1, 2], Qian Jiang [1], Jing Zhang [1, 2, *], Jie Yin [1, 2, *]

[1] Beijing Center for Disease Prevention and Control, Beijing 100013, China

[2] School of Public Health, Capital Medical University, Beijing 100069, China

* Correspondence: brightjing@163.com (Jing Zhang), arnold_jay@163.com (Jie Yin)

Supplementary list

Text S1

Text S2

Table S1−S12

Figure S1

Text S1. Detailed information for tilapia husbandry and exposure in this study

S1.1. Fish characteristics

All tilapia used in this study were adult, sexually mature, commercially farmed *Oreochromis niloticus* with body weights of approximately 1.0 kg, sourced from standardized freshwater fish farms and purchased from a local supermarket in Beijing in 2019. For the intraperitoneal injection experiments described in Sections 2.2 and 3.2, only male individuals were used to preclude any sex-related effects on the metabolism of the target compounds. For the uninjected blank controls, sex was not considered a relevant factor, and thus a mixed-sex population was employed.

S1.2. Sample size

The number of tilapia used in the preliminary intraperitoneal injection experiment (Section 3.2) was determined by the number of experimental groups and the sample size per group. In compliance with the 3R principles to minimize animal use, only two dose levels and three sampling time points were examined, with one dose group corresponding to a single time point. Each group comprised 3 fish, totalling 12 individuals. The data obtained under these conditions were sufficient to reveal the concentration trends of target analytes in fish muscle powders across different doses and time points, providing an adequate basis for subsequent reference material preparation. Given that the accuracy requirements were less stringent than those for dedicated pharmacokinetic studies, no further sample size expansion was undertaken.

For raw material collection, the number of fish was dictated by the total material demand (600 bottles × 5 g/bottle). Taking into account the fillet yield of tilapia (~45%), the dehydration rate of muscle (~80%), and an estimated production loss of approximately 10%, a total of 40 fish were employed, of which 4 were subjected to intraperitoneal injection and the remaining 36 served as untreated blanks.

S1.3. Husbandry and exposure conditions

After being transported to the laboratory within 30 min from the market, all experimental tilapia were acclimated in 60 L water tanks for 12 h without feeding. Five tanks were available in total, and the tanks were thoroughly cleaned after use. Four tanks holding three fish each were used for the preliminary intraperitoneal injection experiment, and one tank housed four fish for formal exposure. The husbandry conditions were as follows:

The water temperature was kept at 24–26 °C, and a photoperiod of 12 hours light and 12 hours dark was adopted. Tap water with aeration pretreatment was applied, and continuous aeration was supplied to maintain dissolved oxygen above 6 mg/L. Given the relatively short holding period for each group of fish, no water exchange was conducted within the 12-hour acclimation, and pH, ammonia-N and nitrite-N concentrations were not monitored. Regular welfare monitoring and clinical observations were conducted during the 12-hour laboratory acclimation and experimental period. Fish were visually inspected every 4 h for abnormal behaviours, body posture, opercular movement and any external lesions. No obvious signs of distress (such as rapid gasping, erratic swimming, loss of balance or lethargy) were observed throughout the whole period. Given the short experimental duration and good health status of all individuals, no fish reached the pre-defined humane endpoints, including severe swimming dysfunction, persistent loss of responsiveness, obvious body injury and inability to maintain normal respiration.

In contrast, blank fish were euthanized immediately after being transported to the laboratory. Acclimation was considered unnecessary for these individuals, as they were only used for raw material collection and did not receive any invasive treatment.

S1.4. Randomization and blinding

Experimental fish were randomly divided into experimental groups with similar body weights to ensure uniform baseline conditions among groups. Group assignment was performed using random numbers generated in Microsoft Excel by a designated operator to minimize subjective bias. Blinding was not applied in the subsequent experiments, since injection and subsequent sample collection were clearly distinguished in chronological order.

Text S2. Equations for single-point quantification, calibration curve fitting, homogeneity assessment, stability assessment and collaborative value assignment

S2.1. The equation for single-point quantification

The mass concentration of bisphenol compounds in fish muscle powder samples was quantified using the single-point calibration method according to the following equation:

$$C_{\text{sample}} = \frac{\mathrm{R_{sample}} \times \mathrm{M_{spike(sample)}} \times \mathrm{M_{calib}}}{\mathrm{R_{calib}} \times \mathrm{M_{spike(calib)}} \times \mathrm{M_{sample}}} \tag{1}$$

where:

C_{sample} represents the concentration of the target analyte in the sample (μg/kg);

$\mathrm{R_{sample}}$ represents the peak area ratio of the target analyte to its isotopically labelled internal standard in the sample solution;

$\mathrm{R_{calib}}$ represents the peak area ratio of the target analyte to its isotopically labelled internal standard in the calibration solution;

$\mathrm{M_{spike(sample)}}$ represents the mass of the isotopically labelled internal standard solution added to the sample (μg);

$\mathrm{M_{spike(calib)}}$ represents the mass of the isotopically labelled internal standard solution added to the calibration solution (μg);

$\mathrm{M_{calib}}$ represents the mass of the native standard of the target analyte in the calibration solution (μg);

$\mathrm{M_{sample}}$ represents the mass of the fish muscle powder test portion (kg).

S2.2. The equation for calibration curve fitting

In order to evaluate the linearity of the quantification method, appropriate volumes of the standard working solution and the mixed internal standard working solution were transferred into 1 mL glass vials and diluted with 50% methanolic water to prepare seven calibration solutions at different concentration levels. The masses of the native standards and the internal standard solution added to each calibration solution were calculated gravimetrically by mass difference. The calibration curves were constructed using the following equation:

$$\frac{A_s}{A_{is}} \times m_{is} = a \times m_s + b \tag{2}$$

where:

A_s is the peak area of the native standard of the target analyte in the calibration solution;

A_{is} is the peak area of the isotopically labelled internal standard in the calibration solution;

m_{is} is the mass of the isotopically labelled internal standard solution added to the calibration solution (μg);

m_s is the mass of the native standard of the target analyte added to the calibration solution (μg);

a is the slope of the calibration curve;

b is the intercept of the calibration curve.

S2.3. The equations for homogeneity assessment

The data obtained in homogeneity studies were analysed by one-way analysis of variance (ANOVA) using the following equations and procedures.

$$v_1 = m - 1 \tag{3}$$

$$v_2 = N - m \ (here\ N = m \times n) \tag{4}$$

$$\bar{x} = \frac{\sum_{j=1}^{n} x_{ij}}{n} \tag{5}$$

$$\bar{\bar{x}} = \frac{1}{N}\sum_{i=1}^{m}\sum_{j=1}^{n} x_{ij} \tag{6}$$

$$Q_1 = \sum_{i=1}^{m} n_i (\bar{x}_i - \bar{\bar{x}})^2 \tag{7}$$

$$Q_2 = \sum_{i=1}^{m}\sum_{j=1}^{n}(x_{ij} - \bar{x}_j)^2 \tag{8}$$

$$MS_{between} = Q_1/v_1 \tag{9}$$

$$MS_{within} = Q_2/v_2 \tag{10}$$

$$F = MS_{between}/MS_{within} \tag{11}$$

Here, v_1 is the degrees of freedom for between bottles, and v_2 is the degrees of freedom for within bottles; n, N and m are the number of replicate measurements per subsample (set as 3), number of total detected samples for the homogeneity study (set as 48) and number of units (set as 16), respectively; x_{ij} is the measured value of the j-th replicate in the i-th bottle; $\bar{x}_i$ is the mean of the i-th bottle; $\bar{x}$ is the mean value of repeated measurements for a single sample; $\bar{\bar{x}}$ is the grand mean over all repeated measurements of all samples; Q_1 is the between-bottle sum of squares; Q_2 is the within-bottle sum of squares; $MS_{between}$ and MS_{within} are the between-bottle and within-bottle mean squares, respectively; and F is the test statistic that follows an F-distribution with degrees of freedom (15, 32).

Based on these degrees of freedom and the pre-defined significance level $\alpha = 0.05$ in this study, the critical value $F_{0.05}(15,32) = 1.992$ is obtained from the F-distribution table. If the calculated F satisfies $F < 1.992$, no significant difference exists between the bottles, and the sample is considered homogeneous. Otherwise, the sample is regarded as non-homogeneous.

S2.4. The equations for stability assessment

The stability of the four target analytes in the fish muscle powder reference material was evaluated using a linear regression method. The storage time was taken as the independent variable (x), and the measured values of the reference material were taken as the dependent variable (y). A linear regression line was fitted according to the following equation, and the corresponding statistical parameters were calculated.

$$\beta_1 = \frac{\sum_{i=1}^{n}[(x_i - \bar{x})(y_i - \bar{y})]}{\sum_{i=1}^{n}(x_i - \bar{x})^2} \tag{12}$$

$$\beta_0 = \bar{y} - \beta_1\bar{x} \tag{13}$$

$$s^2 = \frac{\sum_{i=1}^{n}(y_i - \beta_0 - \beta_1 x_i)^2}{n-2} \tag{14}$$

$$s_\beta = \frac{s}{\sqrt{\sum_{i=1}^{n}(x_i - \bar{x})^2}} \tag{15}$$

where x_i and y_i are the storage time and the corresponding measured value for the i-th time point, respectively; $\bar{x}$ and $\bar{y}$are the means of the storage times and the measured values, respectively; n is the number of time points; β_0 and β_1 are the intercept and slope of the regression line; s^2 is the residual variance, and S_β is the standard error of the slope. The stability was assessed by comparing the absolute value of the slope to its standard error ($|\beta_1|/S_\beta$) against the critical t-value at a 95% confidence level ($\alpha = 0.05$) with $n - 2$ degrees of freedom. If the $|\beta_1|/S_\beta < t_{critical}$, the slope was considered not significantly different from zero, indicating that no significant trend was observed and the material was considered stable under the specified storage conditions.

S2.5. *The equations for the collaborative value assignment*

Outlier detection was carried out using Dixon's test by the following equations and procedures. First, the n replicate measurements in each group were arranged in ascending order: $x_1 \leq x_2 \leq \ldots \leq x_{n-1} \leq x_n$. Second, the Dixon test statistics were calculated as:

$$r_1 = \frac{x_2 - x_1}{x_{n-1} - x_1} \tag{16}$$

$$r_n = \frac{x_n - x_{n-1}}{x_n - x_2} \tag{17}$$

At a significance level of α = 0.05, the critical value r_{critical} (0.05, n) was obtained from the Dixon test table based on the number of replicates n. The following criteria were applied:

If $r_1 > r_n$ and $r_1 > r_{\text{critical}}$, the smallest value x_1 was identified as an outlier and excluded;

If $r_n > r_1$ and $r_n > r_{\text{critical}}$, the largest value x_n was identified as an outlier and excluded;

If both r_1 and r_n were less than r_{critical}, no outliers were detected, and all data points were retained.

Outlier detection was carried out using Grubbs' test by the following equation:

$$G_{calc} = \frac{x - \overline{x}}{s} \tag{18}$$

Where x is the suspect value (either the minimum or maximum); $\overline{x}$ is the arithmetic mean of the measurements; s is the standard deviation of the group; G_{calc} is the Grubbs test statistic. At a significance level of α = 0.05, the critical value $\lambda_{(\alpha,\ n)}$ was obtained from the Grubbs critical value table. The following criteria were applied:

If $G_{calc} > \lambda_{(\alpha, n)}$, the suspect value was identified as an outlier and excluded;

If $G_{calc} < \lambda_{(\alpha, n)}$, no outlier was detected, and all data points were retained.

Cochran's test was used to examine whether the data sets from different groups shared a common variance (i.e., equal precision) and to identify any suspicious data. For m groups, each consisting of n replicate measurements, the variance s_i^2 of each group was first calculated. The Cochran test statistic was then computed as:

$$C = s_{\max}^2 / \sum_{i=1}^{m} s_i^2 \tag{19}$$

Where $s_{\max}^2$ is the largest variance among the m groups, and $\sum_{i=1}^{m} s_i^2$ is the sum of all group variances. At a significance level of α = 0.05, the critical value C_{critical} (α, m, n) was obtained from the Cochran critical value table based on the number of groups (m) and the number of replicates per group (n). The following criteria were applied:

If $C \leq C_{\text{critical}}$ (α, m, n), the group variances were considered homogeneous, indicating equal precision among the data sets, and the results could be combined directly;

If $C > C_{\text{critical}}$ (α, m, n), the largest variance was identified as an outlier, indicating that the precision of that group was significantly poorer than that of the other groups. In this case, the final value should be calculated by taking unequal precision into account .

The normality of all analytical data obtained from eight participating laboratories was assessed using D'Agostino's test. All measurements were arranged in ascending order, and the test statistic Y was calculated according to the following equation:

$$Y = \sqrt{n}\left[\frac{\Sigma\left[\left(\frac{n+1}{2}-K\right)(X_{n+1-K}-X_K)\right]}{n^2\sqrt{m_2}} - 0.28209479\right]/0.02998598 \quad (20)$$

Here, n is the total number of measurements (in this study, n = 64); X_K is the K-th smallest measured value; m_2 is the second central moment, defined as $m_2 = \frac{1}{n}\sum_{i=1}^{n}(x_i - \bar{x})^2$; K ranges from 1 to n / 2 for even n, or from 1 to $(n - 1)/2$ for odd n. The calculated Y statistic was used to test for normality. At a 95% confidence level, the critical interval (Y_{lower}, Y_{upper}) was obtained from the D'Agostino critical value table. If the calculated Y value fell within this interval, the data were considered to follow a normal distribution. Otherwise, the normality assumption was rejected.

Table S1. Concentrations of four analytes in fish muscle powder at different injection doses and exposure times (μg/kg, $n = 3$)

Compounds	Dose Group one			Dose Group two
	3 h *	6 h *	12 h *	12 h *
BPA	259.8 ± 188.3	296.7 ± 43.7	347.8 ± 88.9	90.7 ± 2.6
BPF	151.5 ± 93.0	49.5 ± 29.1	25.0 ± 10.7	64.5 ± 64.8
BPS	68.4 ± 35.4	56.5 ± 25.3	66.6 ± 34.6	70.0 ± 41.5
BPAF	80.0 ± 63.0	109.7 ± 27.0	118.2 ± 23.6	33.8 ± 6.6

Note: Concentrations, expressed as means ± standard deviations calculated using Microsoft Excel, are reported on a freeze-dried fish muscle powder basis ($n = 3$ denotes three independent biological replicates corresponding to three individual fish per experimental group). In Dose Group one, the injection doses were 250 μg/kg, 500 μg/kg, 100 μg/kg and 50 μg/kg for BPA, BPF, BPS and BPAF, respectively. In Dose Group two, the injection doses were 100 μg/kg, 1500 μg/kg, 100 μg/kg and 25 μg/kg for BPA, BPF, BPS and BPAF, respectively. * Sampling times after euthanasia following a single intraperitoneal injection.

Table S2. Linearity and back-calculation results for the calibration curves of four bisphenol analogues

Compounds	Calibration equation	R^2	Theoretical mass (ng)	Back-calculated mass (ng)	Bias (%)	Residual
BPA	$Y = 0.0120694X + 0.00120472$	0.9987	0.8708	0.8128	−6.65	−0.000699
			1.8034	1.7655	−2.10	−0.000458
			2.7361	2.8015	2.39	0.000790
			3.6450	3.6702	0.69	0.000305
			4.6918	4.7648	1.56	0.000881
			5.6388	5.6951	1.00	0.000680
			6.5429	6.4187	−1.90	−0.001499
BPF	$Y = 0.0142912X - 0.000235006$	0.9989	0.9680	0.9894	2.20	0.000305
			2.0048	2.0425	1.88	0.000538
			3.0417	3.0194	−0.73	−0.000318
			4.0520	3.9510	−2.49	−0.001444
			5.2158	5.3022	1.66	0.001234
			6.2685	6.1781	−1.44	−0.001291
			7.2736	7.3419	0.94	0.000976
BPS	$Y = 0.00811921X + 0.000225546$	0.9999	0.9234	0.9112	−1.32	−0.000099
			1.9125	1.9199	0.39	0.000060
			2.9015	2.8968	−0.16	−0.000039
			3.8653	3.8717	0.17	0.000052
			4.9755	5.0111	0.72	0.000289
			5.9797	5.9442	−0.59	−0.000288
			6.9384	6.9414	0.04	0.000024

BPAF	$Y = 0.0153860X - 0.0000385006$	0.9992	0.8727	0.8735	0.11	0.000014
			1.8071	1.8166	0.53	0.000146
			2.7416	2.7141	−1.01	−0.000424
			3.6523	3.6401	−0.33	−0.000188
			4.7013	4.7970	2.04	0.001473
			5.6502	5.5539	−1.70	−0.001482
			6.5561	6.5860	0.46	0.000461

Note: Each calibration curve consisted of seven calibration points. Unweighted linear regression was applied for curve fitting.

Table S3. Homogeneity assessment results for BPA in the RM of fish muscle powder (μ g/kg)

Unit numbers	Measured concentration of three replicates			Means	Standard deviations
	1	2	3		
1	9.08	9.06	9.18	9.11	0.06
2	8.51	8.70	8.99	8.73	0.24
3	8.47	8.84	8.83	8.71	0.22
4	9.34	9.05	9.49	9.29	0.22
5	9.28	8.34	8.88	8.83	0.47
6	9.32	8.73	8.68	8.91	0.35
7	8.91	8.48	8.82	8.74	0.23
8	9.01	8.71	8.99	8.90	0.17
9	9.26	8.94	9.55	9.25	0.30
10	8.85	9.07	8.77	8.90	0.15
11	9.17	9.84	9.10	9.37	0.41
12	9.00	8.98	8.92	8.96	0.04
13	8.53	9.15	9.55	9.07	0.52
14	8.51	9.07	9.30	8.96	0.41
15	8.77	10.18	8.96	9.30	0.77
16	9.33	9.10	8.80	9.08	0.26
overall average				9.01	

Note: Sixteen units were randomly selected for homogeneity assessment, and each unit was measured in triplicate.

Table S4. Homogeneity assessment results for BPF in the RM of fish muscle powder (μg/kg)

Unit numbers	Measured concentration of three replicates			Means	Standard deviations
	1	2	3		
1	7.75	7.22	7.79	7.58	0.32
2	6.94	7.02	6.97	6.98	0.04
3	7.41	7.11	7.48	7.33	0.19
4	7.64	7.19	8.07	7.63	0.44
5	6.98	7.02	7.66	7.22	0.38
6	6.90	7.43	7.21	7.18	0.26
7	7.10	7.43	7.27	7.27	0.17
8	7.56	6.96	7.86	7.46	0.46
9	7.25	7.26	7.11	7.21	0.08
10	7.42	7.20	7.68	7.43	0.24
11	7.46	7.76	7.18	7.47	0.29
12	7.20	7.29	7.52	7.34	0.17
13	7.27	7.20	7.19	7.22	0.04
14	7.05	7.14	7.10	7.10	0.05
15	7.05	7.64	7.68	7.46	0.35
16	7.41	7.42	6.94	7.26	0.27
overall average				7.32	

Note: Sixteen units were randomly selected for homogeneity assessment, and each unit was measured in triplicate.

Table S5. Homogeneity assessment results for BPS in the RM of fish muscle powder (μg/kg)

Unit numbers	Measured concentration of three replicates			Means	Standard deviations
	1	2	3		
1	10.28	10.33	10.25	10.29	0.04
2	10.36	10.63	10.24	10.41	0.20
3	10.20	10.23	10.19	10.21	0.02
4	10.47	10.34	10.84	10.55	0.26
5	10.11	10.07	10.18	10.12	0.05
6	10.29	10.47	10.28	10.35	0.10
7	10.34	10.12	10.16	10.21	0.11
8	10.58	10.20	10.21	10.33	0.22
9	10.16	10.26	10.03	10.15	0.12
10	9.98	10.23	10.20	10.14	0.13
11	10.40	11.24	10.30	10.65	0.51
12	10.05	10.43	10.17	10.22	0.20
13	10.11	10.33	10.43	10.29	0.16
14	10.15	10.01	10.06	10.07	0.07
15	10.05	11.18	10.45	10.56	0.57
16	10.13	10.25	10.27	10.22	0.08
overall average				10.30	

Note: Sixteen units were randomly selected for homogeneity assessment, and each unit was measured in triplicate.

Table S6. Homogeneity assessment results for BPAF in the RM of fish muscle powder (μg/kg)

Unit numbers	Measured concentration of three replicates			Means	Standard deviations
	1	2	3		
1	5.37	5.38	5.31	5.35	0.04
2	5.21	5.45	5.27	5.31	0.13
3	5.41	5.27	5.36	5.34	0.07
4	5.36	5.41	5.48	5.42	0.06
5	5.44	5.26	5.18	5.29	0.13
6	5.40	5.35	5.16	5.30	0.13
7	5.42	5.24	5.11	5.26	0.16
8	5.50	5.33	5.25	5.36	0.13
9	5.09	5.42	5.18	5.23	0.17
10	5.21	5.45	5.26	5.30	0.13
11	5.44	5.93	5.37	5.58	0.30
12	5.25	5.32	5.09	5.22	0.12
13	5.15	5.45	5.12	5.24	0.18
14	5.26	5.33	5.26	5.28	0.04
15	5.31	5.92	5.54	5.59	0.31
16	5.40	5.46	5.42	5.43	0.03
overall average				5.34	

Note: Sixteen units were randomly selected for homogeneity assessment, and each unit was measured in triplicate.

Table S7. Short-term stability test results of bisphenol analytes in the RM of fish muscle powder at 60 °C

Compounds	Monitoring points (days)	Concentrations (μg/kg)				β_1 (95% CI) (μg/kg/day)	ΔC_T (95% CI) (μg/kg)
		Unit 1	Unit 2	Unit 3	Means		
BPA	0	8.52	8.82	8.31	8.74	0.0031	0.031
	1	8.33	8.47	9.06		(−0.0827, 0.0889)	(−0.827, 0.889)
	2	8.62	8.95	8.66			
	4	8.47	8.73	8.95			
	7	9.09	9.05	9.20			
	10	8.88	8.18	8.99			
BPF	0	7.32	7.19	7.39	7.37	−0.0048	−0.048
	1	7.39	7.06	7.33		(−0.0539, 0.0443)	(−0.539, 0.443)
	2	7.41	7.80	7.69			
	4	7.20	7.65	7.44			
	7	7.39	7.30	7.21			
	10	7.17	7.11	7.71			
BPS	0	10.61	10.90	10.62	10.38	0.0257	0.257
	1	10.27	10.23	9.99		(−0.0359, 0.0873)	(−0.359, 0.873)
	2	10.38	10.35	10.28			
	4	9.97	10.11	10.19			
	7	9.80	11.34	10.62			
	10	10.33	10.23	10.68			
BPAF	0	5.40	5.33	5.22	5.17	−0.0093	−0.093
	1	5.16	5.14	5.00		(−0.0401, 0.0215)	(−0.401, 0.215)
	2	5.26	5.23	5.22			
	4	5.06	5.16	5.10			
	7	4.97	5.17	5.11			
	10	5.11	5.10	5.34			

Note: β_1 is the slope of the regression line for each analyte in stability studies; ΔC_T is the predicted change at the endpoint (10 days); 95% CI is the 95% confidence interval of the corresponding value ($\alpha = 0.05$).

Table S8. Long-term stability test results of bisphenol analytes in the RM of fish muscle powder at −20 °C

Compounds	Monitoring points (months)	Concentrations (μg/kg)				β_1 (95% CI) (μg/kg/day)	ΔC_T (95% CI) (μg/kg)
		Unit 1	Unit 2	Unit 3	Means		
BPA	0	8.79	9.28	8.77	8.67	−0.0049	−0.059
	1	8.81	8.38	9.10		(−0.0424, 0.0326)	(−0.509, 0.392)
	3	8.15	8.37	9.17			
	6	8.46	8.92	8.13			
	12	8.17	8.69	8.81			
BPF	0	6.98	7.47	7.11	7.20	0.0018	0.022
	1	7.53	7.25	7.10		(−0.0265, 0.0301)	(−0.318, 0.361)
	3	7.58	7.07	6.84			
	6	7.37	7.02	6.94			
	12	7.44	7.14	7.22			
BPS	0	10.53	10.64	10.46	10.49	−0.0270	−0.324
	1	10.63	10.40	10.79		(−0.0770, 0.0230)	(−0.923, 0.275)
	3	10.43	10.50	10.38			
	6	9.90	10.79	10.34			
	12	10.36	10.39	10.84			
BPAF	0	5.32	5.28	5.16	5.21	−0.0037	−0.044
	1	5.31	5.22	5.22		(−0.0250, 0.0176)	(−0.300, 0.211)
	3	5.18	5.21	5.25			
	6	4.98	5.26	5.09			
	12	5.14	5.17	5.38			

Note: β_1 is the slope of the regression line for each analyte in stability studies; ΔC_T is the predicted change at the endpoint (12 months); 95% CI is the 95% confidence interval of the corresponding value ($\alpha = 0.05$).

Table S9. BPA concentrations in the candidate RM determined by eight laboratories participating in collaborative value assignment (μg/kg).

	Lab 1	Lab 2	Lab 3	Lab 4	Lab 5	Lab 6	Lab 7	Lab 8
1	9.21	8.52	9.10	9.11	8.22	9.25	8.56	8.59
2	8.65	9.26	8.79	8.45	8.31	8.56	8.17	8.63
3	8.95	8.68	8.33	8.50	8.72	8.81	8.37	9.28
4	8.27	8.73	8.94	9.03	8.54	9.28	8.30	8.67
5	8.47	9.05	8.87	8.79	8.23	8.37	8.51	8.19
6	8.74	8.79	8.99	8.92	8.89	8.54	8.71	8.80
7	8.56	8.42	8.40	9.32	8.44	9.02	8.80	8.87
8	8.66	8.32	8.98	9.21	8.58	8.92	8.79	8.39
means	8.69	8.72	8.80	8.92	8.49	8.84	8.52	8.68
standard deviations	0.29	0.31	0.28	0.32	0.24	0.34	0.23	0.33
overall average	8.71							

Table S10. BPF concentrations in the candidate RM determined by eight laboratories participating in collaborative value assignment (μg/kg).

	Lab 1	Lab 2	Lab 3	Lab 4	Lab 5	Lab 6	Lab 7	Lab 8
1	7.67	7.20	7.46	7.54	7.05	7.72	7.28	7.64
2	7.60	7.75	7.22	7.13	7.28	7.26	7.13	7.40
3	7.07	7.27	7.13	7.52	7.55	7.17	7.45	7.31
4	7.20	7.26	7.11	7.72	7.62	7.57	7.40	7.50
5	7.12	7.63	7.42	6.88	7.20	7.12	7.26	7.39
6	7.34	7.05	7.40	7.13	7.48	7.09	7.13	7.15
7	7.25	7.39	7.66	7.31	7.71	7.28	7.22	7.55
8	7.61	7.26	7.38	7.37	7.84	7.14	7.62	7.20
means	7.36	7.35	7.35	7.32	7.47	7.29	7.31	7.39
standard deviations	0.24	0.23	0.19	0.27	0.27	0.23	0.17	0.17
overall average	7.36							

Table S11. BPS concentrations in the candidate RM determined by eight laboratories participating in collaborative value assignment (μg/kg).

	Lab 1	Lab 2	Lab 3	Lab 4	Lab 5	Lab 6	Lab 7	Lab 8
1	10.53	10.58	10.35	10.30	9.89	10.33	9.65	10.02
2	10.63	10.44	10.17	9.83	10.02	10.03	9.77	9.99
3	10.22	10.02	10.00	10.08	10.26	9.81	10.25	10.37
4	9.96	10.18	10.29	10.10	10.45	10.03	9.52	10.15
5	9.77	10.19	10.32	9.83	9.80	9.60	9.86	9.68
6	10.33	10.13	10.35	10.16	10.20	9.79	10.23	10.22
7	9.85	10.22	10.42	10.12	10.11	9.88	9.99	10.01
8	10.14	9.72	10.38	10.17	10.57	10.03	10.56	9.53

means	10.18	10.19	10.29	10.07	10.16	9.94	9.98	10.00
standard deviations	0.31	0.26	0.14	0.16	0.27	0.22	0.35	0.28
overall average				10.10				

Table S12. BPAF concentrations in the candidate RM determined by eight laboratories participating in collaborative value assignment (μg/kg).

	Lab 1	Lab 2	Lab 3	Lab 4	Lab 5	Lab 6	Lab 7	Lab 8
1	5.52	5.67	5.65	5.59	5.32	5.64	5.27	5.48
2	5.62	5.69	5.53	5.33	5.24	5.51	5.22	5.55
3	5.50	5.40	5.30	5.34	5.50	5.32	5.55	5.62
4	5.34	5.45	5.41	5.56	5.48	5.49	5.17	5.55
5	5.22	5.49	5.52	5.20	5.32	5.28	5.27	5.18
6	5.57	5.54	5.58	5.50	5.38	5.24	5.55	5.36
7	5.28	5.54	5.74	5.45	5.30	5.40	5.38	5.35
8	5.44	5.23	5.64	5.49	5.41	5.34	5.71	5.08
means	5.44	5.50	5.55	5.43	5.37	5.40	5.39	5.40
standard deviations	0.14	0.15	0.14	0.13	0.09	0.13	0.19	0.19
overall average				5.43				

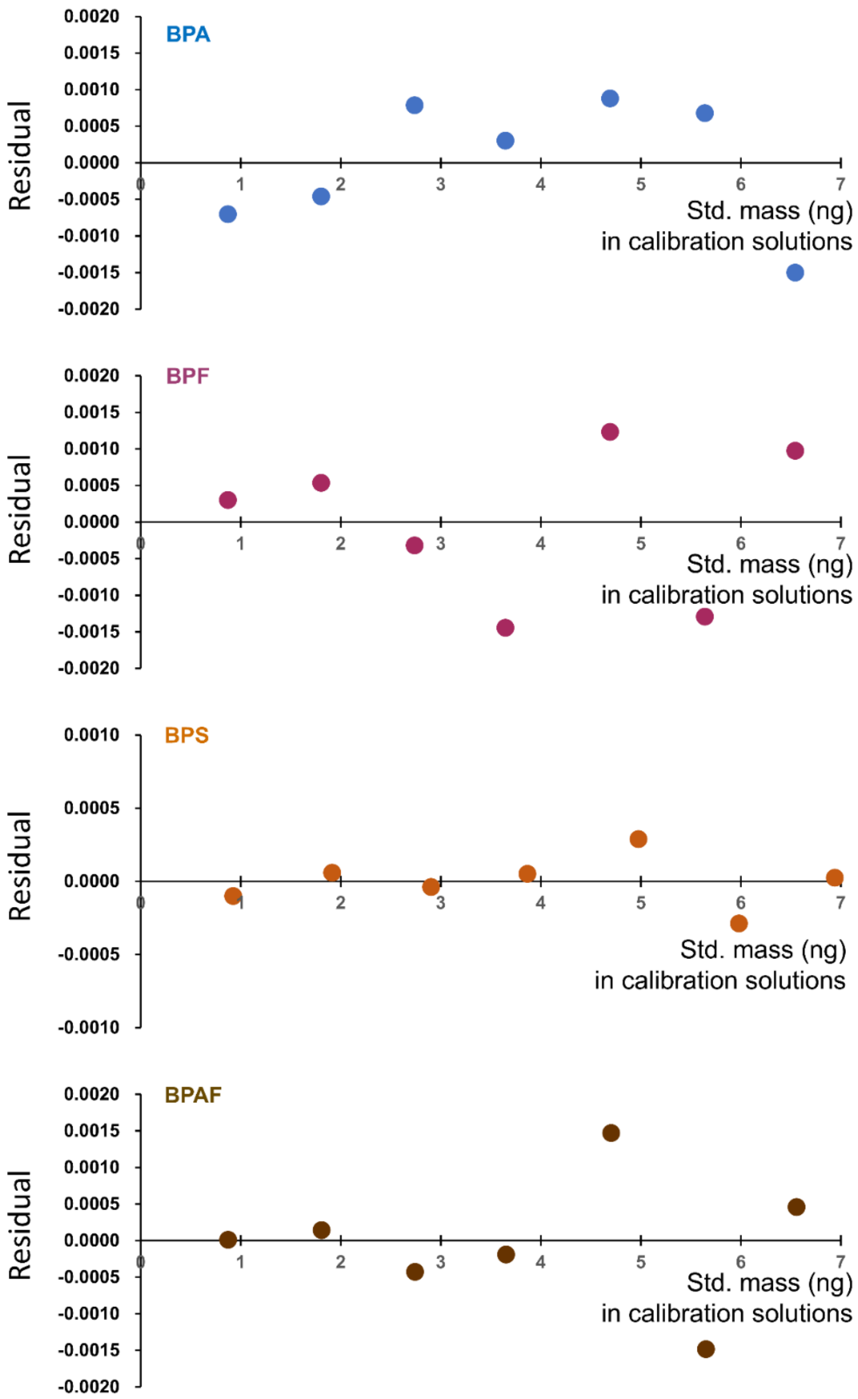

Figure S1. Residual plots for the seven-point calibration curves of BPA, BPF, BPS and BPAF. Residual values are plotted against the standard mass (ng) of each analyte in calibration solutions.
